# Effectively Evaluating a Novel Consensus Subunit Vaccine Candidate to Prevent the H9N2 Avian Influenza Virus

**DOI:** 10.3390/vaccines12080849

**Published:** 2024-07-28

**Authors:** Qi Wu, Weihua Wang, Xuehua Zhang, Ding Li, Mei Mei

**Affiliations:** 1 Institute of Veterinary Immunology & Engineering, Jiangsu Academy of Agricultural Sciences, Nanjing 210014, China; 20210097@jaas.ac.cn (Q.W.); wwh2403@163.com (W.W.); 20100040@jaas.ac.cn (X.Z.); dingli@jaas.ac.cn (D.L.); 2 GuoTai (Taizhou) Center of Technology Innovation for Veterinary Biologicals, Taizhou 225300, China; 3 Jiangsu Key Laboratory of Food and Safety-State Key Laboratory Cultivation Base, Ministry of Science and Technology, Nanjing 210014, China

**Keywords:** H9N2 avian influenza virus, baculovirus expression system, subunit vaccine, hemagglutinin, chicken

## Abstract

The enormous effects of avian influenza on poultry production and the possible health risks to humans have drawn much attention to this disease. The H9N2 subtype of avian influenza virus is widely prevalent among poultry, posing a direct threat to humans through infection or by contributing internal genes to various zoonotic strains of avian influenza. Despite the widespread use of H9N2 subtype vaccines, outbreaks of the virus persist due to the rapid antigenic drift and shifts in the influenza virus. As a result, it is critical to develop a broader spectrum of H9N2 subtype avian influenza vaccines and evaluate their effectiveness. In this study, a recombinant baculovirus expressing the broad-spectrum HA protein was obtained via bioinformatics analysis and a baculovirus expression system (BES). This recombinant hemagglutinin (HA) protein displayed cross-reactivity to positive sera against several subbranch H9 subtype AIVs. An adjuvant and purified HA protein were then used to create an rHA vaccine candidate. Evaluation of the vaccine demonstrated that subcutaneous immunization of the neck with the rHA vaccine candidate stimulated a robust immune response, providing complete clinical protection against various H9N2 virus challenges. Additionally, virus shedding was more effectively inhibited by rHA than by the commercial vaccine. Thus, our findings illustrate the efficacy of the rHA vaccine candidate in shielding chickens against the H9N2 virus challenge, underscoring its potential as an alternative to conventional vaccines.

## 1. Introduction

Avian influenza viruses (AIVs) pose a significant threat to public health, animal welfare, and the global economy [1]. These viruses belong to the Orthomyxoviridae family and are characterized by their eight negative-sense single-stranded RNA segments encoding proteins essential for virulence and adaptation [2]. The viral surface antigenic characteristics, hemagglutinin (HA) and neuraminidase (NA), allow for the classification of influenza viruses into various subtypes [3,4]. Among these subtypes, the H9N2 subtype of AIV poses a significant threat to the poultry industry [5,6]. Initially identified in Wisconsin, USA, in 1966 [7], H9N2 AIV was rapidly disseminated globally, with the first isolation occurring in Guangdong Province, China, in 1994 [8]. Epidemiological studies have indicated a steady increase in H9N2 avian influenza infections since 2016. This subtype has become predominant in China, causing significant harm to chickens, turkeys [9], and waterfowl [10], especially young poultry, and has shown high rates of virus isolation [11]. 

Furthermore, although H9N2 is categorized as a low-pathogenic avian influenza virus (LPAIV), it has a major negative impact on poultry due to its ability to weaken the immune system, increase vulnerability to subsequent illnesses, and cause large financial losses [12]. Notably, large-scale infection with H9N2 in poultry provides a broad gene pool for other subtypes of influenza viruses; H9N2 can widely recombine into H1N1 and H3N2 [13,14], and some internal genes of H9N2 have been shown to enhance the pathogenicity of other subtypes of AIV [15]. There is evidence that the internal genes of the recombinant H7N9 avian influenza virus that broke out in 2013 are directly derived from the H9N2 avian influenza virus [16]. Therefore, H9N2 may be the main potential threat of the next influenza pandemic. In addition, due to the mutation of the HA protein, the binding ability of H9N2 AIV to mammalian upper respiratory tract cells is gradually enhanced [17], and previous studies have shown that H9N2 AIV in poultry populations has acquired the ability to cross the species barrier and directly infect mammals and humans without the need for an intermediate host [18,19]. Therefore, controlling the prevalence of the H9N2 virus, especially in poultry, will reduce the incidence of the H9N2 human infection and reduce the emergence of new recombinant viruses, thus minimizing the risk of an influenza pandemic caused by H9N2 AIV, which is of critical importance to the global poultry industry and human public health [20].

Vaccination remains the most effective strategy for controlling H9N2, but there are significant challenges to the effectiveness of the H9N2 vaccine [21]. With the approval of the initial H9N2 inactivated vaccine in 1998, a range of monovalent and combination vaccines have been utilized in China, significantly contributing to the prevention of H9N2 AIV transmission [22]. There are up to 25 vaccine strains in certified H9 vaccine-related products, all of which are inactivated whole virus vaccines. H9N2 vaccination effectively protects immunized poultry populations by reducing the clinical symptoms produced by viral infections, as evidenced by research and clinical data [20]. As an RNA virus, H9N2 is highly susceptible to antigenic drift and transfer, and inactivated whole-virus vaccines cannot provide adequate protection when the antigenicity of H9N2 vaccine strains is different from that of circulating strains [23], which is one of the challenges of current vaccines against H9N2 [24]. In addition, it should be noted that the inactivated H9N2 vaccine mainly induces humoral immunity, which makes it difficult to block infection and loss of the virus in the upper respiratory tract of chickens [25]. Therefore, the H9N2 AIV strains can carry out effective aerosol transmission between chickens or even between vaccinated chickens [26], which further increases the difficulty of virus prevention and control. Therefore, there is an urgent need to develop a broad-spectrum and effective vaccine for the prevention and control of H9N2 AIVs to adapt to the current multiantigen coepidemic situation.

Subunit vaccinations have become a focus of contemporary vaccine research due to their good safety and immunogenicity [27]. Recombinant protein vaccines are safer than live virus-derived vaccines due to their nonreplicative nature and lack of infectious components [28]. The baculovirus expression system, widely used for producing recombinant protein vaccines, significantly reduces production costs [29]. In silico methods of bioinformatics and immunoinformatics can be used for more rapid and precise vaccine design [30,31]. As the main surface antigen of influenza virus, HA protein is responsible for binding and entering host cells [3], inducing neutralizing antibodies, and stimulating the body to produce cellular immunity [32]. Therefore, genetically engineered vaccines based on HA proteins have shown good immunogenicity and promising applications [33].

In this study, a recombinant baculovirus expressing the HA protein was designed by combining bioinformatics analysis with a BES. CVCVA5, an adjuvant that can activate immune cells, was added to the main immunogenic antigen of the recombinant protein vaccine to improve its protective effect [34]. The effectiveness of the rHA vaccine candidate against the avian influenza virus was evaluated.

## 2. Materials and Methods

### 2.1. Cells and Viruses

Sf9 insect cells (ATCC, Manassas, VA, USA) were cultured in Grace’s Insect Medium (Gibco, Carlsbad, CA, USA), while High Five (Hi5) cells (ATCC, USA) were cultured in SF900II Medium (Invitrogen, Waltham, MA, USA). The challenge viruses used in the study included three LPAI strains originating from clade h9.4.2.5 A/Chicken/Zhejiang/2017 (115) and A/Chicken/Jiangsu/YZLH (YZLH), as well as clade h9.4.2.3 A/Chicken/Shandong/6 (SD/6). Ten positive serum samples (115, 236, YZLH, SD/6, and Wisconsin/1) were prepared and stored in our laboratory according to the methods described previously [34]. Three strains originated from clade h9.4.2.5, while the SD/6 strain and Wisconsin/1 strain originated from clades h9.4.2.3 and h9.1, respectively.

### 2.2. Generation of HA Gene-Based Recombinant Protein

The consensus HA sequence of the H9 subtype was determined in this study by aligning all available complete HA sequences of the H9 subtype isolated from avian species in China between 2017 and 2021. These sequences were retrieved from the Influenza Research Database, GenBank, and the Global Initiative on Sharing Avian Influenza Data. The alignment was performed using the Clustal V program within the Lasergene software suite. The consensus HA sequence was generated by selecting the most frequently occurring nucleotides at each position across these sequences. Subsequently, the codons in the resulting recombinant HA (rHA) were optimized to match the codon usage preferences of *S. frugiperda* cells, a modification carried out by Tsingke Biotechnology (Nanjing, China). Once optimized, the consensus HA gene was synthesized by the same institution.

After synthesis, the consensus HA gene was inserted into the pFastBac-1 vector. The next step involved creating a recombinant bacmid by transforming the purified plasmid DNA into DH10Bac-competent cells and utilizing a blue/white selection process for identification. The confirmed recombinant bacmid was then transfected into Sf9 cells to produce recombinant baculoviruses (rBVs) using the TransIT-LT1 transfection reagent (Mirusbio, Madison, WI, USA). The genomic DNA of these rBVs was extracted and verified through PCR analysis. After successful verification, the rBVs were transfected into Sf9 cells, and an indirect immunofluorescence assay (IFA) was subsequently performed to validate the rescue effect of the rBVs.

The rescued rBVs were serially reproduced in High Five cell suspension culture via eight passages after three rounds of plaque purification in Sf9 cells. To evaluate the expression of HA proteins in the rBVs, Sf9 cells were cultured in 6-well plates and infected with the rBVs at an MOI of 1. At 96 h postinfection, the infected High Five cells were harvested and lysed via ultrasonication for 10 min at 4 °C. The resulting cell lysates were then further purified through sucrose density gradient centrifugation. HA protein expression was confirmed through Western blot analysis. Furthermore, a hemagglutination assay (HA) was used to test the functionality of the rHA protein, and a BCA Protein Assay Kit (Vazyme, Nanjing, China) was utilized to measure the protein concentration.

### 2.3. Production of the rHA Vaccine Candidate

The purified rHA proteins were concentrated to their initial volume before being subjected to ultracentrifugation in PBS at a pH of 7.2. Each dose contained approximately 50 µg of rHA mixed with the mineral oil and adjuvant CVCVA5, as described in our previous report [34]. Briefly, the components of CVCVA5 consisted of an oil phase including imiquimod and resiquimod added to mineral oil, as well as an aqueous phase containing poly(I:C), levamisole hydrochloride, and the L-D isoform muramyl dipeptide added to the rHA antigen solution. Three volumes of an oil phase were combined with one volume of an aqueous phase. The concentration of the rHA antigen in the stock solutions was standardized to ensure that the vaccine preparations had HA titers of at least 256. After undergoing characterization, stability, and sterility assessments, the sterile rHA vaccine candidate was dispensed into vaccine vials and stored at 4 °C.

### 2.4. Chicken Vaccination Experiments and Viral Challenge Study

A cohort of 117 10-day-old specific-pathogen-free (SPF) chickens was divided into three groups, each consisting of 18 individuals. Group 1, labeled as the rHA vaccine candidate group, received a subcutaneous injection of the rHA vaccine candidate in the neck at a dose of 0.5 mL per chicken. Group 2, known as the commercial vaccine group, was given a commercial vaccine at a dose of 0.3 mL per chicken. The commercial vaccine used was an inactivated avian influenza vaccine (H9 subtype, clade h9.4.2.5 HN03 strain) purchased from a vaccine company in China. The third group of unvaccinated chickens served as the negative control group. Serum samples were collected at 2, 3, and 4 weeks postimmunization to isolate the serum for subsequent determination of hemagglutination inhibition (HI) antibody titers.

At 28 d after immunization, the chickens were challenged with three H9 subtype AIV epidemic strains, 115, YZLH, and SD/6, which belong to different subbranches, at a concentration of 10^6.5^ EID_50_ per chicken. Daily observations of feed and water intake, as well as the general health status of the chickens, were conducted to detect any abnormalities. Incidence rates and mortality were also documented.

### 2.5. Body Weights and Histopathology

The weights of the chickens in each challenge group were recorded on days 2, 4, 6, 8, 10, 12, and 14 post-challenge to monitor weight changes and calculate the relative weight gain rate for each group. Three chickens in each group were euthanized at 5 dpi, and lung samples were collected and subjected to hematoxylin and eosin (HE) staining for pathomorphological analysis.

### 2.6. Viral Shedding

Cloacal and oropharyngeal swabs were obtained from all groups on days 3, 5, and 7 post-challenge. Subsequently, these swabs were inoculated into 10-day-old SPF chicken embryos, with each sample receiving 0.2 mL of swab fluid. Any chicken embryos that died within the first 24 h were excluded, and the HA titer of the allantoic fluid from surviving embryos was measured after 120 h. Briefly, initially, a 25 µL allantoic fluid was subjected to serial dilution by a factor of 2 in a 96-well plate filled with PBS. Next, 25 µL of 1% chicken red blood cells was added, and the mixture was incubated for an additional 25 min at room temperature. The HA titer was then calculated as the reciprocal of the highest dilution preventing red blood cell agglutination after the 25-min incubation period. A chicken was considered to have cleared the virus if the HA titer in either the throat or cloacal swab-derived allantoic fluid was ≥2 log2. A result <2 log2 indicated a suspicious case requiring passage through a blind generation for further verification. Chickens were classified as negative, signifying no viral shedding if the HA titer remained <2 log2 after the blind passage. Meanwhile, viral RNA extraction from swabs was conducted with an AxyGEN RNA kit (AXYGEN, Hangzhou, China) in strict adherence to the manufacturer’s guidelines. The identification primer set, which was specifically designed to target the M gene of the H9N2 avian influenza virus, consisted of the forward primer 5′-GCGTAGACGGTTTGTC-3′ and the reverse primer 5′-TCCGCAATCTGCTCACAA-3′. The RT-qPCR was performed with ChamQ SYBR Color qPCR Master Mix (Vazyme, Nanjing, China), and the data were analyzed using an ABI QuantStudio 5 Q5 real-time PCR system (Thermo Fisher, Waltham, MA, USA).

### 2.7. HI Assay

Serum samples were collected every 7 days postimmunization for the detection of hemagglutination inhibition (HI) antibodies using standardized procedures. Initially, a 25 µL serum sample was subjected to serial dilution by a factor of 2 in a 96-well plate filled with PBS. Subsequently, 25 µL of AIV antigen (4 units) was added, and the mixture was incubated at room temperature for 25 min to facilitate the binding of antigen–antibody complexes. Next, 25 µL of 1% chicken red blood cells was added, and the mixture was incubated for an additional 25 min at room temperature. The HI antibody titer was then calculated as the reciprocal of the highest dilution preventing red blood cell agglutination after the 25-minute incubation period. 

### 2.8. Statistical Analysis

Data analysis was performed using GraphPad Prism version 10.0 software. Data were presented as the mean values ± SE of at least 3 replicates, unless otherwise indicated. Statistical significance was analyzed by either *t*-tests or one-way analysis of variance (ANOVA), followed by Dunn’s multiple comparisons test. All *p* values of <0.05 were considered statistically significant. Asterisk markers for significance levels: * for *p* < 0.05, ** for *p* < 0.01, and *** for *p* < 0.001.

## 3. Results

### 3.1. Successful Rescue and Characterization of rHA 

**Figure 1:**
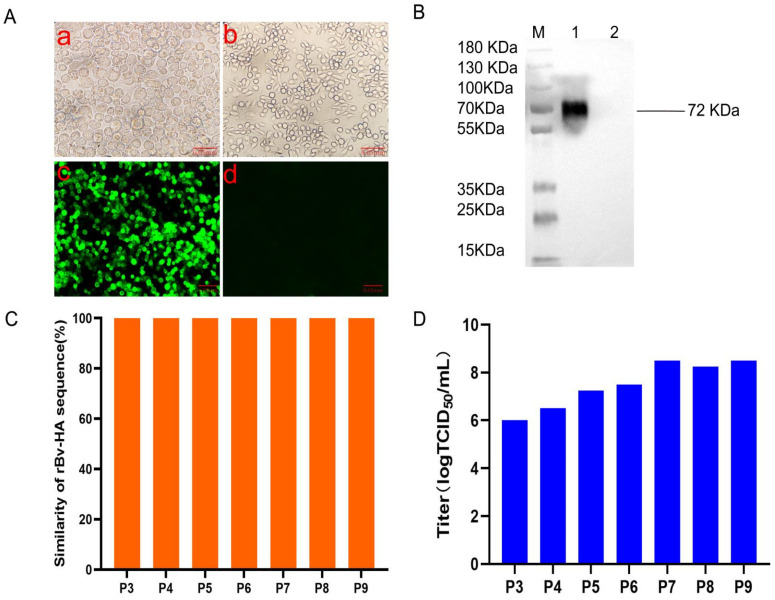
Characterization of the H9 AIV recombinant HA protein. (**A**) The expression of the recombinant proteins was analyzed by IFA with a polyclonal antibody against H9 AIV. Light microscopy was used to observe Sf9 cells infected with recombinant baculovirus rBV-HA (a) and normal Sf9 cells (b); IFA was analyzed in Sf9 cells infected with recombinant baculoviruses (rBVs) of HA (c) or only empty baculoviruses (d) after 72 h. (**B**) The purity of the rHA protein was analyzed using Western blot analysis with a polyclonal antibody against H9 AIV. (**C**) The stability of the recombinant baculovirus was confirmed through sequencing analysis. (**D**) The viral titer of recombinant baculovirus passages was determined using the plaque formation method.

A recombinant baculovirus (rBv-HA) that expresses the HA protein was successfully generated and described in this study. Upon transfection into Sf9 cells, noticeable cellular morphological changes, such as increased cell size, rounding, and growth arrest, which are typical signs of viral infection, were observed (Figure 1A). Indirect immunofluorescence analysis revealed that cells infected with rBv-HA baculovirus exhibited robust specific green fluorescence; in contrast, cells infected with wild-type baculovirus displayed only minimal non-specific fluorescence (Figure 1A). This result confirmed the generation of the recombinant virus. 

In further studies, rBv-HA was used to infect suspended High Five cells. The cell culture medium collected 96–120 h postinfection was subjected to high-pressure disruption. Western blot analysis revealed a distinct band at 72 kDa in all samples, indicating the good antigenicity of the rHA protein (Figure 1B). The hemagglutination titer of rHA was assessed using 1% chicken red blood cells and reached 12 log2, suggesting that the biological activity of rHA is comparable to that of natural HA in agglutinating red blood cells. Sequence determination from the first to nineth generations of the rHA baculovirus confirmed that the HA gene sequence was consistent with the target sequence, demonstrating excellent genetic stability (Figure 1C). Notably, starting from the fifth generation, the virus titer remained consistently high (TCID_50_ > 8 log10), highlighting the superior reproductive performance of the recombinant baculovirus (Figure 1D).

### 3.2. rHA Protein Displays Cross-Reactivity to Positive Sera against H9 Subtype AIVs

**Figure 2:**
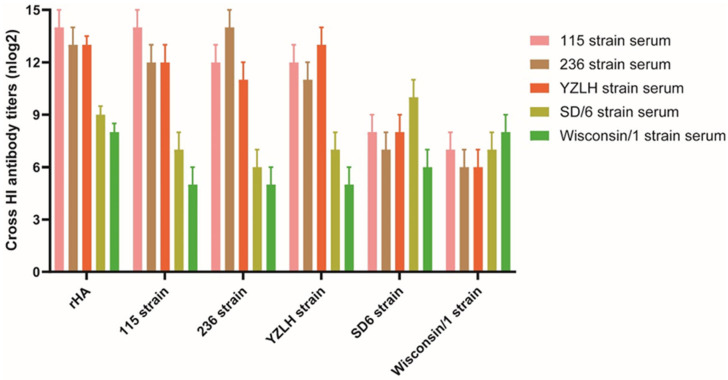
Cross-HI results of different subbranched strains of H9 subtype AIV and the recombinant protein HA. Three strains (115, 236, and YZLH) originated from clades h9.4.2.5, and the SD/6 and Wisconson/1 strains originated from clades h9.4.2.3 and h9.1, respectively.

HI assays were used to determine the antigenicity of the rHA protein and to measure its cross-reactivity with sera against different subbranch H9 AIVs. The results illustrated that the rHA displayed robust reactivity with sera from various H9 subtypes of AIV and exhibited cross-reactivity with diverse strains, such as SD/6 and Wisconsin/1, emphasizing its strong cross-reactive nature and indicating that the rHA protein holds promise for providing broad protection against a range of H9 avian influenza subtypes, underscoring its value as a versatile and effective immunogen for combating avian influenza (Figure 2).

### 3.3. The rHA Subunit Vaccine Induces Potent Immune Responses 

**Figure 3:**
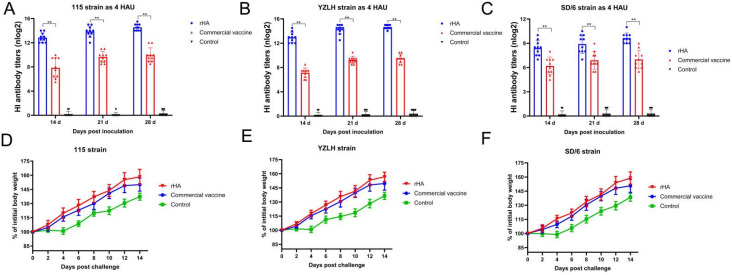
Immune response and protective efficacy of the rHA vaccine candidate in chickens. (**A**) The hemagglutination inhibition (HI) antibody titers of the immune sera were assessed using the 115-strain antigen. (**B**) The HI antibody titers of the immune sera were determined with the YZLH strain antigen. (**C**) The HI antibody titers of the immune sera were measured using the SD/6 strain antigen. (**D**) The body weight gain rate of chickens challenged with the 115 strain was monitored every 2 d for 14 d. (**E**) The body weight gain rate of chickens challenged with the YZLH strain was recorded every 2 d for 14 d. (**F**) The body weight gain rate of chickens challenged with the SD/6 strain was assessed every 2 d for 14 d. Statistical significance was calculated by a one-way ANOVA. ** *p* < 0.01. The data represent the mean values and error bars for SEM.

SPF chicks aged 10 days were given either the commercial vaccine or the rHA vaccine candidate to evaluate the vaccine immunogenicity. Serum antibody levels against various antigens were determined using the HI assay at 14, 21, and 28 days post-vaccination. When four HA units (HAU) of strain 115 were used as the testing antigen, chickens vaccinated with the rHA vaccine candidate exhibited higher HI titers than those induced by the commercial vaccine (Figure 3A). Similarly, the HI titers elicited by the rHA vaccine candidate surpassed those induced by the commercial vaccine when the YZLH strain was used as the 4-HAU testing antigen (Figure 3B). Furthermore, when the SD/6 strain was used as the 4-HAU antigen, the HI titer induced by the rHA vaccine candidate surpassed that induced by the commercial vaccine (Figure 3C). These findings demonstrated the high immunogenicity of the rHA vaccination. 

### 3.4. The rHA Subunit Vaccine Provides Significant Protection against the H9 Subtype AIV Challenge 

To assess the protective effect of the vaccine, SPF chickens were immunized for 4 weeks and then challenged with various subbranches of the H9 AIV strains (115, YZLH, and SD/6) at 10^6.5^ EID_50_, with 13 SPF chickens in each group. Clinical observations revealed no fatalities within 14 d post-challenge. The rHA group and commercial vaccine group showed no abnormalities post-challenge, with normal feed and water intake and stable mental states. In contrast, the control group exhibited reduced appetite and thirst during the initial 4 d following the challenge. At 2, 4, 6, 8, 10, 12, and 14 d post-challenge, the chickens in each group underwent weight measurements, and the relative growth rate of their body weight was calculated. The results revealed that the vaccinated group had a much greater rate of weight growth than the challenged control group, with the rHA group displaying the greatest relative weight gain rate (Figure 3D–F). Notably, after the SD/6 challenge, the weight gain of chickens immunized with the rHA vaccine candidate significantly differed from that of the control group. Compared with that in the commercial vaccine group, the weight gain in the group immunized with the rHA vaccine candidate was greater than that in the commercial vaccine group, although the difference was not significant.

**Figure 4:**
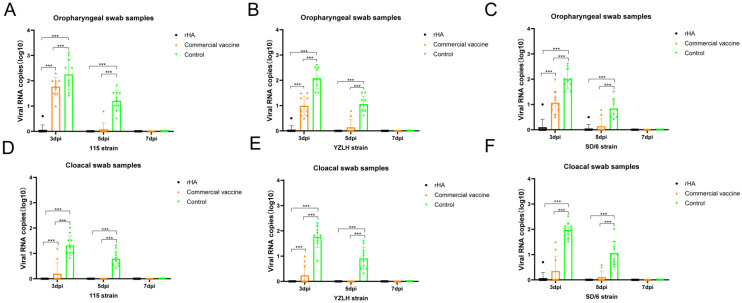
Virus shedding from the immunized chicken after the H9N2 virus challenge. The viral shedding of the 115 strain was evaluated in oropharyngeal (**A**) and cloacal (**D**) swabs by RT-qPCR. YZLH strain viral shedding was evaluated in oropharyngeal (**B**) and cloacal (**E**) swabs by RT-qPCR. The viral shedding of the SD/6 strain was evaluated in oropharyngeal (**C**) and cloacal (**F**) swabs by RT-qPCR. The statistical significance of the differences in HI titers was analyzed with a *t*-test. The error bars represent the standard deviation. Comparisons used to generate *p* values are indicated by horizontal lines (*** *p* < 0.001).

To further investigate the protective efficacy of the two vaccines against H9 AIV, oropharyngeal and cloacal swabs were obtained and analyzed at 3, 5, and 7 d post-challenge. Three days post-challenge, a notable difference in viral shedding emerged between the vaccinated groups. Specifically, all ten chickens in the commercial vaccine cohort exhibited virus shedding, whereas only a single chicken in the rHA-vaccinated group exhibited this viral shedding (Figure 4A–F). The results of the challenge with 115 strains revealed that no viruses were detectable in oropharyngeal or cloacal swabs at day 5 in the rHA vaccine candidate group (Figure 4A,D). In contrast, one oropharyngeal swab sample from the commercial vaccine group tested positive for AIV at day 5, while all swab samples from the challenged control group were positive for AIV. 

Similarly, in the rHA vaccine candidate group, none of the immunized chickens exhibited viral shedding at day 5 of the YZLH strain challenge, whereas two out of ten chickens in the commercial vaccine group and all five chickens in the challenged control group shed the virus (Figure 4B,E). Furthermore, following the SD/6 strain challenge, one oropharyngeal swab sample from the rHA vaccine candidate group showed viral shedding. Two out of the ten chickens shed the virus in the commercial vaccine group (Figure 4C,F), while all chickens in the challenged control group exhibited viral shedding (Table 1).

**Figure 5:**
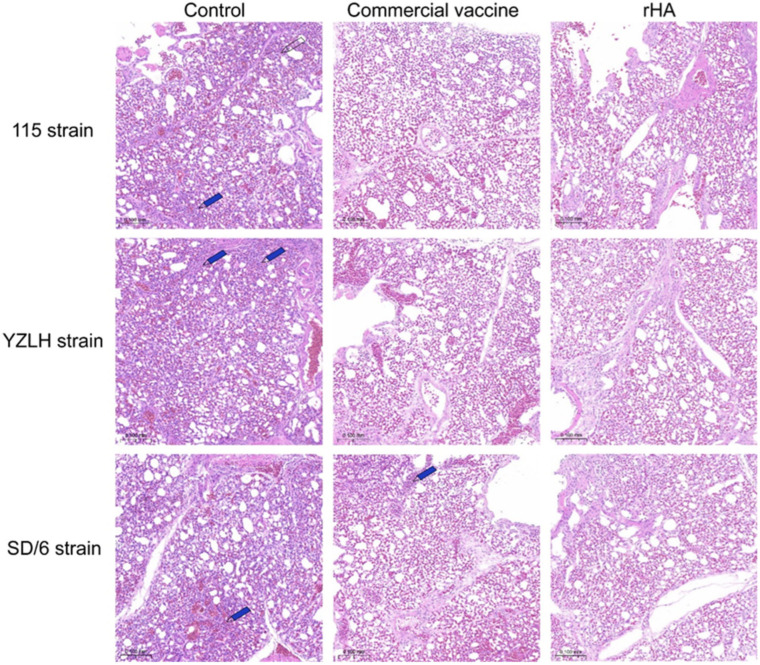
Histopathological examination of the lungs of challenged chickens at 5 d p.c. The 115 strain, YZLH strain, and SD/6 strain originated from clade h9.4.2.5 and clade h9.4.2.3, respectively. The scale bar is 0.100 mm. The blue arrows represent inflammatory cell infiltration.

AIV tends to replicate primarily in the lungs, leading to tissue damage. To assess the potential role of the rHA vaccine candidate in mitigating virus-induced lung damage, histopathological alterations in the lungs were examined using HE staining. The commercial vaccination group demonstrated mild inflammatory cell infiltration, while the rHA vaccine candidate group showed no significant pathological alterations. These findings suggest that the rHA vaccine candidate is a good one (Figure 5).

## 4. Discussion

H9N2 infection impairs the immune function of poultry and increases susceptibility to secondary diseases, resulting in significant economic losses [35]. Vaccination remains the main method for controlling H9N2 avian influenza in China [36]. The use of inactivated vaccines can reduce the clinical symptoms of H9N2 infection and minimize economic losses [37]. H9N2 avian influenza viruses are highly susceptible to antigenic drift and transfer, and inactivated whole-virus vaccines cannot provide adequate protection when the antigenicity of the H9N2 vaccine strain is different from that of the circulating strain [26,38], which is one of the current challenges of vaccination against H9N2. In recent years, with the progress of bioinformatics technology, we have gained a deeper understanding of the pathogenic mechanism of influenza virus infection [31]. It is possible to design and develop vaccines with broad-spectrum protection against H9N2 and other AIV subtypes.

The creation of a universal AIV vaccine that can trigger a widespread immune response is essential for reducing the spread of the disease. The HA protein of the influenza virus has undergone continuous evolution to evade both natural infections and vaccine-induced herd immunity while maintaining its essential functions [39]. HA, a major envelope glycoprotein, mediates viral attachment to host cells by binding to sialic acid receptors, facilitating viral entry and the subsequent release of mature virions [40]. It attaches itself to sialic acid molecules on the surface of respiratory tract epithelial cells, allowing the virus to cling to them [2]. Comprising two main components, the head and the stalk, the head contains the receptor-binding domain that aids in virus recognition and attachment to specific host-cell receptors [23]. The virus is able to adhere to respiratory tract epithelial cells by attaching itself to sialic acid molecules on its surface [1]. Traditionally, the HA head is known for its ability to induce neutralizing antibodies and serves as the primary target of current influenza vaccines. Recent discoveries of broadly neutralizing antibody epitopes on the HA stalk have offered valuable insights into the structure-based design of immunogen [41]. The importance of concentrating on this domain to construct a broad-spectrum influenza vaccine that provides broad protection against a variety of influenza strains is highlighted by the discovery of HA stalk immunogens that can elicit heterosubtypic antibodies [42]. The discovery and analysis of multiple broadly neutralizing antibodies directed at HA have paved the way for developing structure-based universal influenza vaccine candidates [43]. In this study, bioinformatics was used to analyze the HA protein sequence binding of H9 subtype avian influenza strains from 2017 to 2021 and obtain a consensus HA sequence. A recombinant baculovirus expressing the HA protein was successfully constructed using a BES. The evaluation of the recombinant HA protein showed that the rHA protein had good cross-reactivity with different H9 subbranch AIV-positive sera. The rHA protein antigen lays the groundwork for a proposed broad-spectrum subunit vaccine candidate.

To evaluate the efficacy of the rHA protein, a subunit vaccine candidate was made by augmentation with adjuvants to enhance the stimulation of an effective immune response. This subunit vaccine candidate vaccination of chickens has produced encouraging findings in terms of eliciting a strong antibody-mediated immune response and eliciting sera that cross-react with antigens from different H9 subtypes. Notably, the results of viral shedding detection on the fifth day after the challenge demonstrated that the rHA vaccine candidate more effectively blocked the oral shedding of various subbranch H9 subtypes of AIV than did the commercial vaccine (Table 1). Recently, studies have shown that the H9AIV vector vaccine and subunit vaccine have a good immune protection effect, and the protective efficacy is comparable to that of the inactivated vaccine [27,33]. However, the efficacy of broad-spectrum protection remains to be verified. The results of this study indicate that bioinformatics technology in the design of a broad-spectrum H9AIV vaccine antigen that specifically targets HA has proven efficacious. However, the broad-spectrum rHA vaccine candidates need to be further optimized. Consequently, additional optimization studies are needed to determine the broad-spectrum protective effectiveness of the rHA vaccine candidate by incorporating more broad-spectrum AIV epitopes and adjuvants into the antigen design.

As a promising technology for vaccine manufacturing, BES provides a strategically significant vaccine platform [29,44]. It is valued for its high safety, rapid production, and adaptable product design. Baculovirus-mediated protein folding and glycosylation lead to the production of proteins with natural conformation and biological activity, thereby enhancing the immunogenicity and protective efficacy of vaccines [45]. As a result, the expressed HA protein closely resembles the original HA protein and has good antigenicity and immunogenicity. This approach guarantees effective and stable expression of the HA protein while maintaining its native structure and function. Hu et al. demonstrated that influenza virus-like particles derived from baculoviruses offer complete protection against the lethal H7N9 avian influenza virus challenge in chickens and mice [44]. Notably, mosaic influenza virus-like particle vaccines produced using a BES elicit a broad humoral and cellular immune response to influenza A virus [46]. This study utilized a baculovirus expression system to achieve high-titer, stable expression of recombinant HA protein. The HA protein expressed by Sf9 cells had a molecular weight of approximately 72 kDa (Figure 1D), significantly exceeding the expected 60 kDa. This finding demonstrated that the Sf9 cell line can help foreign proteins undergo glycosylation changes. This study revealed that the recombinant baculovirus exhibited an excellent titer (TCID_50_ > 8 log10), demonstrating robust genetic stability. 

In conclusion, a method that combines BES with bioinformatics research resulted in the development of a broad-spectrum subunit H9 vaccine candidate. Immunization with the rHA vaccine candidate induced a robust HI antibody response, ensuring 100% clinical protection against various strains of the H9N2 virus in chickens. Furthermore, rHA more effectively suppressed viral shedding and significantly reduced lung lesions in chickens than did the commercial vaccine, highlighting the potential of the rHA vaccine candidate as an alternative to conventional avian influenza vaccines for controlling H9 avian influenza infections in poultry.

## Figures and Tables

**Table 1:** Virus shedding from immunized chickens after H9N2 virus challenge.

Challenge Virus	Vaccine	Oropharyngeal Swabs			Cloacal Swabs		
		3 d	5 d	7 d	3 d	5 d	7 d
h9.4.2.5 clade of H9 AIV (115 strain)	rHA	1/10	0/10	0/10	0/10	0/10	0/10
	Commercial	10/10	1/10	0/10	2/10	0/10	0/10
	Control	10/10	10/10	0/10	10/10	10/10	0/10
h9.4.2.5 clade of H9 AIV (YZLH strain)	rHA	1/10	0/10	0/10	0/10	0/10	0/10
	Commercial	10/10	2/10	0/10	3/10	0/10	0/10
	Control	10/10	10/10	0/10	10/10	10/10	0/10
h9.4.2.3 clade of H9 AIV (SD/6 strain)	rHA	1/10	1/10	0/10	1/10	0/10	0/10
	Commercial	10/10	2/10	0/10	3/10	2/10	0/10
	Control	10/10	10/10	0/10	10/10	10/10	0/10

## Data Availability

The data presented in this study are available in this article.

## References

[1] Li Y.T., Linster M., Mendenhall I.H., Su Y.C.F., Smith G.J.D. (2019). Avian influenza viruses in humans: Lessons from past outbreaks. Br. Med. Bull..

[2] Lycett S.J., Duchatel F., Digard P. (2019). A brief history of bird flu. Philos. Trans. R. Soc. Lond. B Biol. Sci..

[3] Carnaccini S., Perez D.R. (2020). H9 Influenza Viruses: An Emerging Challenge. Cold Spring Harb. Perspect. Med..

[4] Gu M., Xu L., Wang X., Liu X. (2017). Current situation of H9N2 subtype avian influenza in China. Vet. Res..

[5] Bhat S., James J., Sadeyen J.R., Mahmood S., Everest H.J., Chang P., Walsh S.K., Byrne A.M.P., Mollett B., Lean F. (2022). Coinfection of Chickens with H9N2 and H7N9 Avian Influenza Viruses Leads to Emergence of Reassortant H9N9 Virus with Increased Fitness for Poultry and a Zoonotic Potential. J. Virol..

[6] Pusch E.A., Suarez D.L. (2018). The Multifaceted Zoonotic Risk of H9N2 Avian Influenza. Vet. Sci..

[7] Homme P.J., Easterday B.C. (1970). Avian influenza virus infections. I. Characteristics of influenza A-turkey-Wisconsin-1966 virus. Avian Dis..

[8] Jackwood M.W., Yousef N.M.H., Hilt D.A. (1997). Further development and use of a molecular serotype identification test for infectious bronchitis virus. Avian Diseases.

[9] Fellahi S., Nassik S., Maaroufi I., Tligui N.S., Touzani C.D., Rawi T., Delvecchio A., Ducatez M.F., El Houadfi M. (2021). Pathogenesis of Avian Influenza Virus Subtype H9N2 in Turkeys and Evaluation of Inactivated Vaccine Efficacy. Avian Diseases.

[10] Li X., Sun J., Lv X., Wang Y., Li Y., Li M., Liu W., Zhi M., Yang X., Fu T. (2020). Novel Reassortant Avian Influenza A(H9N2) Virus Isolate in Migratory Waterfowl in Hubei Province, China. Front. Microbiol..

[11] Bi Y., Li J., Li S., Fu G., Jin T., Zhang C., Yang Y., Ma Z., Tian W., Li J. (2020). Dominant subtype switch in avian influenza viruses during 2016-2019 in China. Nat. Commun..

[12] Yang W., Liu X., Wang X. (2023). The immune system of chicken and its response to H9N2 avian influenza virus. Vet. Q..

[13] Sorrell E.M., Wan H.Q., Araya Y., Song H.C., Perez D.R. (2009). Minimal molecular constraints for respiratory droplet transmission of an avian-human H9N2 influenza A virus. Proc. Natl. Acad. Sci. USA.

[14] Sun Y.P., Qin K., Wang J.J., Pu J.A., Tang Q.D., Hu Y.X., Bi Y.H., Zhao X.L., Yang H.C., Shu Y.L. (2011). High genetic compatibility and increased pathogenicity of reassortants derived from avian H9N2 and pandemic H1N1/2009 influenza viruses. Proc. Natl. Acad. Sci. USA.

[15] Peng C., Zhao P., Chu J., Zhu J., Li Q., Zhao H., Li Y., Xin L., Yang X., Xie S. (2022). Characterization of four novel H5N6 avian influenza viruses with the internal genes from H5N1 and H9N2 viruses and experimental challenge of chickens vaccinated with current commercially available H5 vaccines. Transbound. Emerg. Dis..

[16] Lam T.T., Wang J., Shen Y., Zhou B., Duan L., Cheung C.L., Ma C., Lycett S.J., Leung C.Y., Chen X. (2013). The genesis and source of the H7N9 influenza viruses causing human infections in China. Nature.

[17] Lloren K.K.S., Lee T., Kwon J.J., Song M.S. (2017). Molecular Markers for Interspecies Transmission of Avian Influenza Viruses in Mammalian Hosts. Int. J. Mol. Sci..

[18] Um S., Siegers J.Y., Sar B., Chin S., Patel S., Bunnary S., Hak M., Sor S., Sokhen O., Heng S. (2021). Human Infection with Avian Influenza A(H9N2) Virus, Cambodia, February 2021. Emerg. Infect. Dis..

[19] Zhang B.S., Li L.J., Zhu Q., Wang Z., Yuan P., Zhou G.D., Shi W.J., Chu X.F., Jiang S.J., Xie Z.J. (2020). Co-infection of H9N2 influenza virus and contributes to the development of hemorrhagic pneumonia in mink. Vet. Microbiol..

[20] Liu S., Zhuang Q., Wang S., Jiang W., Jin J., Peng C., Hou G., Li J., Yu J., Yu X. (2020). Control of avian influenza in China: Strategies and lessons. Transbound. Emerg. Dis..

[21] Dong J.Z., Zhou Y., Pu J., Liu L.T. (2022). Status and Challenges for Vaccination against Avian H9N2 Influenza Virus in China. Life.

[22] Yu X., Jin T., Cui Y., Pu X., Li J., Xu J., Liu G., Jia H., Liu D., Song S. (2014). Influenza H7N9 and H9N2 viruses: Coexistence in poultry linked to human H7N9 infection and genome characteristics. J. Virol..

[23] Kim Y.H., Hong K.J., Kim H., Nam J.H. (2022). Influenza vaccines: Past, present, and future. Rev. Med. Virol..

[24] Liu Y., Zhao D., Zhang J., Huang X., Han K., Liu Q., Yang J., Zhang L., Li Y. (2023). Development of an Inactivated Avian Influenza Virus Vaccine against Circulating H9N2 in Chickens and Ducks. Vaccines.

[25] Xu S., Zhang B., Yao J., Ruan W. (2023). A new H9 influenza virus mRNA vaccine elicits robust protective immunity against infection. Vaccine.

[26] Zhang N., Quan K., Chen Z., Hu Q., Nie M., Xu N., Gao R., Wang X., Qin T., Chen S. (2023). The emergence of new antigen branches of H9N2 avian influenza virus in China due to antigenic drift on hemagglutinin through antibody escape at immunodominant sites. Emerg. Microbes Infect..

[27] Zhu S., Nie Z., Che Y., Shu J., Wu S., He Y., Wu Y., Qian H., Feng H., Zhang Q. (2024). The Chinese Hamster Ovary Cell-Based H9 HA Subunit Avian Influenza Vaccine Provides Complete Protection against the H9N2 Virus Challenge in Chickens. Viruses.

[28] Bissett S.L., Roy P. (2024). Multi-Gene Recombinant Baculovirus Expression Systems: From Inception to Contemporary Applications. Viruses.

[29] Hong Q., Liu J., Wei Y., Wei X. (2023). Application of Baculovirus Expression Vector System (BEVS) in Vaccine Development. Vaccines.

[30] Ishack S., Lipner S.R. (2021). Bioinformatics and immunoinformatics to support COVID-19 vaccine development. J. Med. Virol..

[31] Ullah A., Waqas M., Aziz S., Rahman S.U., Khan S., Khalid A., Abdalla A.N., Uddin J., Halim S.A., Khan A. (2023). Bioinformatics and immunoinformatics approach to develop potent multi-peptide vaccine for coxsackievirus B3 capable of eliciting cellular and humoral immune response. Int. J. Biol. Macromol..

[32] Caceres C.J., Rajao D.S., Perez D.R. (2021). Airborne Transmission of Avian Origin H9N2 Influenza A Viruses in Mammals. Viruses.

[33] Pan X., Liu Q., Niu S., Huang D., Yan D., Teng Q., Li X., Beerens N., Forlenza M., de Jong M.C.M. (2022). Efficacy of a recombinant turkey herpesvirus (H9) vaccine against H9N2 avian influenza virus in chickens with maternal-derived antibodies. Front. Microbiol..

[34] Wu P., Lu J., Zhang X., Mei M., Feng L., Peng D., Hou J., Kang S.M., Liu X., Tang Y. (2017). Single Dose of Consensus Hemagglutinin-Based Virus-Like Particles Vaccine Protects Chickens against Divergent H5 Subtype Influenza Viruses. Front. Immunol..

[35] Xu C., Ye H., Qiu W., Lin H., Chen Y., Zhang H., Liao M. (2018). Phylogenetic classification of hemagglutinin gene of H9N2 avian influenza viruses isolated in China during 2012-2016 and evaluation of selected candidate vaccine strains. Poult. Sci..

[36] Xia J., Li Y.X., Dong M.Y., Guo Z.W., Luo Y.W., Li N.L., Zhao Y., Li M., Lin Y., Xu J. (2023). Evolution of prevalent H9N2 subtype of avian influenza virus during 2019 to 2022 for the development of a control strategy in China. Poult. Sci..

[37] Wei K., Li Y. (2018). Global genetic variation and transmission dynamics of H9N2 avian influenza virus. Transbound. Emerg. Dis..

[38] Wang X., Liu K., Guo Y., Pei Y., Chen X., Lu X., Gao R., Chen Y., Gu M., Hu J. (2023). Emergence of a new designated clade 16 with significant antigenic drift in hemagglutinin gene of H9N2 subtype avian influenza virus in eastern China. Emerg. Microbes Infect..

[39] Song C.L., Liao Z.H., Shen Y., Wang H., Lin W.C., Li H., Chen W.G., Xie Q.M. (2020). Assessing the efficacy of a recombinant H9N2 avian influenza virus-inactivated vaccine. Poult. Sci..

[40] Shi J., Zeng X., Cui P., Yan C., Chen H. (2023). Alarming situation of emerging H5 and H7 avian influenza and effective control strategies. Emerg. Microbes Infect..

[41] Sayedahmed E.E., Elkashif A., Alhashimi M., Sambhara S., Mittal S.K. (2020). Adenoviral Vector-Based Vaccine Platforms for Developing the Next Generation of Influenza Vaccines. Vaccines.

[42] Park J., Fong Legaspi S.L., Schwartzman L.M., Gygli S.M., Sheng Z.M., Freeman A.D., Matthews L.M., Xiao Y., Ramuta M.D., Batchenkova N.A. (2022). An inactivated multivalent influenza A virus vaccine is broadly protective in mice and ferrets. Sci. Transl. Med..

[43] Guthmiller J.J., Han J., Utset H.A., Li L., Lan L.Y., Henry C., Stamper C.T., McMahon M., O’Dell G., Fernandez-Quintero M.L. (2022). Broadly neutralizing antibodies target a haemagglutinin anchor epitope. Nature.

[44] Hu J., Zhang Q., Peng P., Li R., Li J., Wang X., Gu M., Hu Z., Hu S., Liu X. (2022). Baculovirus-derived influenza virus-like particle confers complete protection against lethal H7N9 avian influenza virus challenge in chickens and mice. Vet. Microbiol..

[45] Chen J., Wang J., Zhang J., Ly H. (2021). Advances in Development and Application of Influenza Vaccines. Front. Immunol..

[46] Liu X., Zhao T., Wang L., Yang Z., Luo C., Li M., Luo H., Sun C., Yan H., Shu Y. (2023). A mosaic influenza virus-like particles vaccine provides broad humoral and cellular immune responses against influenza A viruses. NPJ Vaccines.

